# Breast Milk Vitamin C Concentrations and Their Association With Confinement Dietary Practices in the Early Postpartum Period

**DOI:** 10.7759/cureus.103305

**Published:** 2026-02-09

**Authors:** Nurul Husna Mohd Shukri, Mawarni Abdullah, Nuruljannah Mohamad Nasri, Siti Raihanah Shafie, Jonathan C.K. Wells, Mary Fewtrell

**Affiliations:** 1 Department of Nutrition, Faculty of Medicine and Health Sciences, Universiti Putra Malaysia, Serdang, MYS; 2 Childhood Nutrition Research Centre, UCL Great Ormond Street Institute of Child Health, London, GBR

**Keywords:** ascorbic acid, breastfeeding, confinement practices, human milk, maternal diet

## Abstract

Vitamin C is a potent antioxidant that supports both short- and long-term health in infants. As breast milk is the main source of vitamin C for infants, maternal postpartum dietary practices may influence vitamin C concentrations in breast milk and thereby affect infant intake. This study aimed to examine the trend of vitamin C levels in breast milk during the postpartum confinement period in Malaysian mothers and their association with maternal dietary intake. It was designed as a longitudinal observational study using data from a randomised controlled trial investigating relaxation therapy. Sixty-four first-time mothers in the Klang Valley participated. Breast milk samples were collected during home visits at week 2 and weeks 8-12 postpartum. Vitamin C content in breast milk was measured using high-performance liquid chromatography. Participants completed a questionnaire covering sociodemographic characteristics and traditional postpartum practices, including a food list that assessed changes in dietary intake (classified as avoided, reduced, maintained, or increased) at two weeks postpartum. The mean vitamin C concentration in breast milk was 3.87 ± 2.8 µg/mL, which was lower than values commonly reported in other populations. A significant positive correlation was observed between vitamin C concentrations at the early and later time points (r=0.60, p=0.022). However, vitamin C levels were significantly lower at week 2 than at weeks 8-12 (p=0.04). At 2 weeks postpartum, fruit intake remained largely unchanged, whereas eggs and nuts were the most frequently avoided foods. Conversely, fish and vegetable consumption increased the most. Fruit intake correlated with vitamin C at week 2 (r=0.573, p=0.01), but not later, and vitamin C was not associated with other food categories (p>0.05). These findings highlight the importance of adequate intake of vitamin C-rich foods (e.g., fruits) during confinement to support breast milk levels, reinforcing the importance of culturally sensitive dietary guidance in maternal care. Nevertheless, generalizability may be limited because participants were predominantly educated urban Malay mothers.

## Introduction

Breast milk is the gold standard of infant feeding, providing essential nutrients and bioactive components that support both short- and long-term health outcomes in infants. Among the key micronutrients required during infancy is vitamin C, a potent antioxidant that plays several critical biological roles. It supports immune function by scavenging free radicals and maintaining the integrity of natural barriers against infection [1]. Vitamin C also enhances the absorption of non-heme iron, thereby contributing to the prevention of iron deficiency anaemia in infants [2]. Breast milk is an important source of vitamin C for infants, but its concentration could be influenced by maternal diet and nutritional status.

Studies have shown that breast milk vitamin C concentrations vary over time and between populations. A recent longitudinal study among Chinese mothers reported dynamic changes in breast milk vitamin content, including vitamin C, across colostrum, transitional, and mature milk stages [3]. Among Bangladeshi mothers, vitamin C concentrations decreased from colostrum to mature milk [4]. In contrast, Martysiak-Żurowska et al. (2016) found stable vitamin C levels within a single feeding session among Polish mothers [5]. These findings highlight inconsistencies in the temporal pattern of breast milk vitamin C, likely influenced by population differences, maternal dietary intake, or methodological variations. To date, no published data are available on this topic among Malaysian mothers, highlighting a gap in the local literature.

Vitamin C concentration in breast milk is influenced by maternal dietary intake, as confirmed by systematic reviews from Bravi et al. (2016) reporting positive associations with dietary and supplemental intake contributing to breast milk levels [6]. This highlights the need for lactating mothers to meet vitamin C requirements to support both their health and breast milk quality. According to the Malaysian Recommended Nutrient Intakes (RNI), lactating women require 95 mg of vitamin C per day, which is slightly higher than the 85 mg/day recommended by the World Health Organization [7]. However, a study by Agustina et al. (2023) reported that many Malay mothers consistently fell below the recommended intake throughout the breastfeeding period [8]. This raises concerns about potential inadequacies in breast milk vitamin C content and related infant health outcomes.

The low intake of vitamin C may be attributed to dietary restrictions associated with traditional postpartum confinement practices. In Malaysia, such practices are widely observed across Malay, Chinese, and Indian communities and typically include adherence to food taboos, postpartum massage, and the use of traditional herbs. The average duration of confinement is approximately 40 to 44 days for Malay mothers and 30 days for Chinese and Indian mothers [9]. Dietary practices, particularly food taboos, form a key component of confinement and vary significantly across cultural groups.

Restrictions on foods high in vitamin C during the confinement period may influence its concentration in breast milk. In Malay and Chinese cultures, certain fruits and vegetables are avoided due to their perceived "cold" or "sharp" properties, such as watermelon, cucumber, and pineapple [9,10]. Among Malaysian Indian mothers, tomatoes and cucumbers are typically avoided, while green leafy vegetables are encouraged to promote breast milk production [9]. A similar trend has been observed in Sudan, where 19% of 165 breastfeeding mothers reported avoiding vitamin C-rich foods such as onion, garlic, radish, spices, and legumes due to cultural beliefs [11]. These findings suggest that food taboos may lead to reduced intake of vitamin C-rich foods during confinement; however, their impact on breast milk vitamin C concentration among Malaysian mothers has not been previously reported.

Hence, there is a concern that traditional confinement diets among Malaysian mothers may influence the nutritional quality of breast milk, particularly its vitamin C concentration. To date, no published studies have examined the trend of vitamin C levels in the breast milk of Malaysian mothers or their association with postpartum dietary intake. Therefore, this study aimed to examine changes in breast milk vitamin C concentrations between two time points (week 2 and weeks 8-12 postpartum) and their associations with maternal dietary intake during confinement. Understanding this relationship may help inform culturally appropriate dietary guidance for lactating mothers during confinement and support public health efforts to optimise breast milk micronutrient quality.

## Materials and methods

Study design

This was a longitudinal observational study utilising data from the MOMStudy, a randomised controlled trial (ClinicalTrials.gov identifier: NCT01971216) investigating relaxation therapy and mother-infant signalling among exclusively breastfeeding mothers [12]. Exclusive breastfeeding status was confirmed using screening questions from the MOMStudy follow-up questionnaire administered at regular intervals (every two to four weeks postpartum). Ethical approval was obtained from both the Medical Research Ethics Committee (MREC), Ministry of Health Malaysia (ID: 13-841-16720), and the University College London (UCL) Ethics Committee (ID: 4883).

Data collection

This study included a subset of breast milk samples collected from the MOMStudy, which involved 64 first-time mothers residing in the Klang Valley, Malaysia. Of these, 35 mothers had sufficient breast milk volume (≥1 mL) at both week 2 and weeks 8-12 postpartum for high-performance liquid chromatography (HPLC) vitamin C analysis and were therefore included in the analysis. Furthermore, this sample size is comparable to those used in previous breast milk vitamin C studies and was deemed sufficient for exploratory analyses of trends and associations. During home visits, the samples were initially stored in milk storage containers placed in insulated boxes containing frozen silica pads. After each visit, the samples were transferred into 15 ml tubes and stored at -80°C until laboratory analysis. In addition, mothers completed a self-administered questionnaire covering sociodemographic characteristics, maternity care, and postpartum traditional practices. They reported who provided care during the postpartum period for both mother and infant, the duration of confinement, and the extent of adherence to traditional practices. Dietary practices were assessed using a food list (Appendix A) in which mothers indicated changes in their intake of specific food categories during the confinement period, classified as avoided, reduced, maintained, or increased [13]. The reported changes are relative to their usual pre-confinement diet.

Breast milk sample preparation

Breast milk samples were prepared for vitamin C analysis following the protocol by Romeu-Nadal et al. [14]. Once samples were collected during the home visit, they were stored in a cold, insulated box to minimise degradation. Samples were transferred to −80°C storage within a few hours of collection. Prior to analysis, the tubes were wrapped in aluminium foil to protect the samples from light-induced degradation. Samples stored in 15 ml tubes were thawed at approximately 22°C for 30 minutes in a water bath. A 2 ml aliquot of each sample was then pipetted for analysis. For reagent preparation, stock standard solutions were prepared by dissolving ascorbic acid in 0.56% (w/v) meta-phosphoric acid using Milli-Q water (Milli-Q®, Merck Millipore, Burlington, MA, USA) and stored at 4°C. The mobile phase Milli-Q water was purified using a Millipore Compact Milli-Q water system (Merck Millipore). Analytical-grade reagents included standard ascorbic acid (99.7% purity) and meta-phosphoric acid (33.5%-36.5% purity). HPLC-grade acetic acid and methanol were used as solvents in the analysis.

Determination of vitamin C

Vitamin C content in breast milk was determined using HPLC, adapted from the method described by Romeu-Nadal et al. [14]. Briefly, 600 µL of breast milk was mixed with 600 µL of 0.56% (w/v) meta-phosphoric acid, shaken for 30 seconds, and centrifuged at 10°C for 10 minutes at 3000 rpm. A 50 µL aliquot of the resulting filtrate was directly injected into the HPLC system. Isocratic chromatographic separation was performed using a mobile phase consisting of Milli-Q water with 0.1% (v/v) acetic acid and methanol in a ratio of 95:5 (v/v), delivered at a flow rate of 0.7 mL/min. The column temperature was maintained at 25°C. Ascorbic acid was identified by comparing the retention time of sample peaks with that of an ascorbic acid standard, with detection at 254 nm. Vitamin C in breast milk was quantified using a standard calibration curve of ascorbic acid (0.5-6 µg/ml), based on the linear equation y = 24.382x + 1.9604 (R² = 0.998). All breast milk samples were analysed in duplicate, and the mean value was used for statistical analysis.

Statistical analysis

All data were entered and analysed using IBM SPSS Statistics software, version 22 (IBM Corp., Armonk, NY, USA). Categorical variables were presented as frequencies and percentages, while continuous variables were reported as means and standard deviations. Changes in vitamin C concentrations between early and later home visits were assessed using the Wilcoxon signed-rank test due to the non-normal distribution of the data. Spearman’s rank correlation coefficient was used to assess the correlation between vitamin C concentrations at the two time points and to assess the association between maternal dietary intake changes (classified as avoided, reduced, maintained, or increased) and vitamin C concentrations in breast milk. A p-value of less than 0.05 was considered statistically significant.

## Results

Sociodemographic characteristics

Most mothers (38, 59.4%) were aged between 26 and 30 years, with a mean age of 26.66 ± 2.8 years. The majority were Malay (60, 93.8%) and had attained at least a bachelor’s degree (39, 60.9%). Regarding household income, 19 (29.7%) of mothers reported earnings between Ringgit Malaysia (RM) 1500-RM3000, and another 19 (29.7%) between RM5001-RM8000. The average duration of confinement was 43.25 ± 10.4 days, with most mothers (44, 84.6%) practising confinement for 30 to 50 days. Detailed sociodemographic characteristics of the participants are presented in Table 1.

**Table 1:** Sociodemographic characteristics of mothers *1 USD = RM4.2; RM: Ringgit Malaysia

Characteristic	N%
Age range (years)	
20-25	21 (32.8)
26-30	38 (59.4)
31-34	5 (7.8)
Ethnicity	
Malay	60 (93.8)
Chinese	2 (3.1)
Others	2 (3.2)
Educational level	
Primary school	1 (1.6)
Secondary school	9 (14.1)
Certificates/Diploma	8 (12.5)
Bachelor degree	39 (60.9)
Postgraduate	7 (10.9)
Household income*	
RM1500-RM3000	19 (29.7)
RM3000-RM5000	16 (25.0)
RM5001-RM8000	19 (29.7)
>RM8001	10 (15.7)
Confinement duration	
< 30 days	5 (9.6)
30 - 50 days	44 (84.6)
> 50 days	3 (5.8)

Vitamin C concentration in breast milk

Mean breast milk vitamin C concentrations were 2.69 ± 2.2 µg/mL at week 2 and 4.25 ± 2.9 µg/mL at weeks 8-12, with an overall mean of 3.87 ± 2.8 µg/mL across all samples, which is markedly lower than concentrations commonly reported in other populations (approximately 30-50 µg/mL). A significant positive correlation was observed between vitamin C concentrations at week 2 and weeks 8-12 (r=0.60, p=0.022). However, paired analyses showed that vitamin C levels were significantly lower at week 2 than at weeks 8-12 (Wilcoxon signed-rank test, p = 0.041) (Table 2).

**Table 2:** Trend of changes and correlation of vitamin C (µg/mL) across time-points * Wilcoxon signed-rank test

Data	Week 2	Weeks 8-12
Mean ± SD (µg/mL)	2.69 ± 2.2	4.25 ± 2.9
Paired t-test * (p- value)	0.041	
Correlation result (r-value, p-value)	0.60, 0.022	

Maternal dietary intake

**Figure 1:**
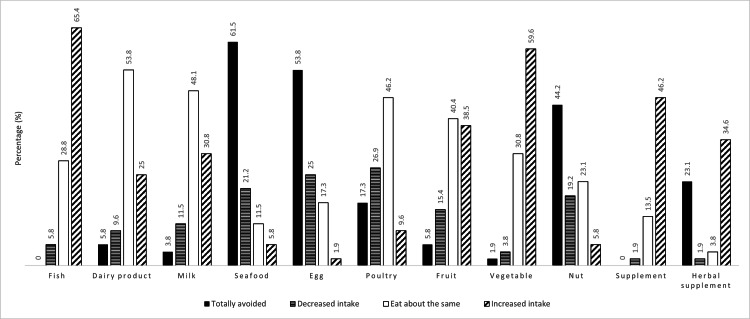
Changes in maternal dietary intake across food items during postpartum confinement

As shown in Figure 1, mothers reported varying changes in intake across different food categories during the postpartum confinement period. The top three foods most commonly avoided were seafood/shellfish (n=32, 61.5%), eggs (n=28, 53.8%), and nuts (n=23, 44.2%). Poultry showed the highest proportion of mothers who reduced their intake (n=14, 26.9%), followed by eggs (n=13, 25.0%) and seafood/shellfish (n=11, 21.2%). In contrast, the largest proportions of mothers who increased their intake were reported for fish (n=34, 65.4%) and vegetables (n=31, 59.6%). The majority of mothers reported maintaining (n=21, 40.4%) or increasing their fruit intake (n=20, 38.5%), with reduced or avoided intake observed in a small proportion.

Association of vitamin C concentration in breast milk with changes in food intake

As shown in Table 3, there were no significant associations between breast milk vitamin C concentrations and maternal intake of most food items during the confinement period, except for fruits. A strong positive correlation was observed between fruit intake and vitamin C concentration in breast milk at the early time point (r=0.57, p=0.01). However, no significant associations were found between fruit intake and vitamin C concentrations at the later time point (p>0.05). This pattern reflects the timing of dietary assessment at week 2 and suggests that early postpartum breast milk vitamin C levels are sensitive to maternal vitamin C intake, with fruits being a key dietary source.

**Table 3:** Correlations between maternal dietary intake patterns during early postpartum confinement and breast milk vitamin C concentrations at week 2 and weeks 8-12 postpartum Note. Dietary intake was assessed at week 2 and categorised as avoided, reduced, maintained, or increased (ordinal scale). Correlations were computed using Spearman’s rank correlation coefficient (rs). Significant values (p < 0.05) are shown in bold*

Food items	Vitamin C Week 2		Vitamin C Weeks 8-12	
	rs	p-value	rs	p-value
Milk	-0.01	0.982	-0.08	0.672
Dairy	-0.001	0.982	-0.11	0.560
Poultry	-0.37	0.124	0.02	0.911
Egg	0.12	0.625	-0.10	0.580
Fish	0.38	0.111	-0.19	0.294
Seafood	-0.09	0.713	-0.11	0.551
Nuts	-0.19	0.430	0.07	0.712
Fruits	0.57	0.010*	0.10	0.591
Vegetables	0.01	0.967	-0.34	0.062
Tablet supplement	0.26	0.347	0.19	0.371
Herbal supplement	0.09	0.781	0.22	0.356

## Discussion

This study provides important insight into breast milk vitamin C concentrations among Malaysian mothers, highlighting the influence of traditional postpartum dietary practices. The average vitamin C concentration across all collected breast milk samples was 3.87 ± 2.8 µg/mL, which is substantially lower than values reported in many other populations (approximately 30-50 µg/mL). For example, studies in Germany and Japan using similar HPLC methods reported mean concentrations of 50.9 ± 16.5 µg/ml and 51.0 ± 19 µg/ml, respectively [5,15]. In Bangladesh, spectrophotometric analysis showed concentrations ranging from 30.3 to 35.2 µg/ml throughout lactation [4]. This discrepancy may be due to differences in dietary intake, nutritional status, sampling time, and analytical methods [3-5]. Although samples in this study were stored at -80°C, in line with standard recommendations, the four-year storage period could have affected vitamin C stability. Previous studies indicate a degradation of approximately 15.0% in vitamin C content after one year or more of storage at −80°C [16]. Even after accounting for this expected degradation, the adjusted concentrations in the present study remain substantially lower than those reported in other populations, and this adjustment does not materially alter the overall interpretation of the findings. Despite this limitation, these findings offer important insight into how culturally influenced dietary practices during confinement may relate to breast milk vitamin C concentrations among Malaysian mothers. Moreover, the use of HPLC ensured reliable quantification throughout the analysis.

In this study, breast milk vitamin C concentrations showed a significant increase from week 2 to weeks 8-12 postpartum. This differs from findings by Martysiak et al. (2016), who reported no significant variation in vitamin C levels across lactation stages, suggesting a potential physiological threshold for secretion [5]. In contrast, other studies have documented a declining trend. A recent longitudinal study among Chinese mothers reported dynamic changes in breast milk vitamin content, including vitamin C, across colostrum, transitional, and mature milk stages [3]. Similarly, Ahmed et al. (2004) reported a decline from 35.2 ± 5.6 µg/mL in colostrum to 30.3 ± 6.7 µg/mL in mature milk among Bangladeshi mothers [4]. These mixed findings across studies indicate that temporal patterns of vitamin C concentration in breast milk are not uniform and may vary depending on maternal nutritional status, dietary intake, analytical methods, or timing of sample collection. Notably, although concentrations increased over time in the present study, vitamin C levels at week 2 were strongly correlated with levels at weeks 8-12, suggesting that early postpartum concentrations are associated with later values, possibly reflecting individual physiological regulation of vitamin C secretion [5].

In terms of overall dietary patterns, this study found that seafood was the most commonly avoided or reduced food item during confinement, followed by eggs, nuts, and poultry. This is consistent with findings by Yahya et al. (2023), who also reported avoidance of seafood, eggs, black pepper, and coffee among postpartum Malaysian women [10]. Such practices are shaped by cultural beliefs that certain foods may cause itchiness, “toxicity,” or delayed wound healing [17,18]. In many Southeast Asian settings, seafood is broadly restricted except for snakehead fish (*Channa striatus*), which is traditionally perceived to promote postoperative and perineal wound healing [19]. Scientific evidence supports some of these beliefs, as snakehead fish may enhance leukocyte production and tissue repair and reduce inflammation [19,20]. Egg avoidance was also commonly reported in this study, although evidence from Indonesia suggests that egg consumption during postpartum recovery can aid wound healing [21]. Importantly, current guidelines do not recommend the avoidance of allergenic foods such as eggs and fish during lactation, as maternal dietary restriction does not prevent food allergies in infants [22]. Although these foods are not major sources of vitamin C, their avoidance reflects broader confinement-related dietary restrictions that may reduce overall dietary variety, which could contribute to the low breast milk vitamin C concentrations observed in this study.

In contrast to avoided foods, mothers in this study reported increased consumption of fish, vegetables, and vitamin supplements during the confinement period. This trend aligns with findings from Singapore among Chinese, Malay, and Indian mothers, who reported similar shifts in dietary intake during the postpartum period [23]. Traditionally, Malay confinement practices restricted certain foods based on beliefs that some fish may be toxic and that vegetables have “cold” properties that could slow recovery [13]. The increased intake observed in this cohort may reflect evolving nutritional awareness and a gradual relaxation of restrictive dietary taboos [24]. Because fish and vegetables are important dietary sources of vitamin C and other micronutrients, their increased intake after completion of confinement may support maternal nutrition and could help explain the higher vitamin C concentrations at weeks 8-12, when most mothers had already completed confinement, compared to week 2.

Regarding vitamin C-rich foods specifically, avoidance rates in this study were much lower than previously reported. Only 5.8% and 1.9% of mothers completely avoided fruits and vegetables, respectively, whereas a larger proportion reported maintaining or increasing intake. This contrasts with earlier studies, such as Poh et al. (2005), which found that over 70.0% of mothers avoided vegetables and nearly half avoided fruits during confinement [18] due to beliefs about “cold,” “sharp,” or gas-producing properties [9]. Similar beliefs have been documented in Bangladesh, where postpartum women restricted vegetables, pumpkin, and certain fruits to prevent infant diarrhoea [25]. Reduced fruit and vegetable intake can compromise maternal vitamin C status, which is essential for tissue repair, immune function, and antioxidant protection during postpartum. The relatively low avoidance observed in the present study likely contributed to the moderate breast milk vitamin C concentrations seen at weeks 8-12, although levels remained low compared to international values. Inadequate vitamin C intake during infancy has been linked to increased vulnerability to infection and poor antioxidant protection. As breast milk is the sole source of vitamin C for exclusively breastfed infants, low milk vitamin C concentrations may warrant attention, although infant vitamin C status and health outcomes were not assessed in this study.

Importantly, this study found that increased fruit intake during early confinement was significantly associated with higher breast milk vitamin C concentrations at week 2, but not at weeks 8-12. This pattern is consistent with the timeline of postpartum practices, as confinement typically lasts 30-50 days and dietary restrictions diminish thereafter. The findings align with previous research demonstrating that breast milk vitamin C is sensitive to maternal dietary intake. Martysiak et al. (2016) reported that vitamin C derived from natural food sources was more efficiently transferred to breast milk than vitamin C from supplements [5], and Bravi et al. (2016) similarly identified dietary intake, rather than supplementation, as the primary determinant of its concentration in breast milk [6]. Consistent with the present findings, no association was observed between supplement use and breast milk vitamin C levels.

Overall, the study findings indicate that although many Malaysian mothers continue to follow confinement dietary practices, several restrictions, particularly those involving fruits and vegetables, appear to be declining. Nevertheless, restrictive confinement diets still pose nutritional risks. Given the importance of adequate nutrient intake for postpartum recovery and breast milk quality, antenatal education encouraging balanced and culturally acceptable dietary practices remains essential. Evidence from a randomised trial by Liu et al. (2009) suggests that antenatal nutrition counselling can reduce dietary taboos and improve intake during confinement [26]. Because most mothers in this study observed confinement for 30-50 days, similar to practices across Asian cultures [9, 27], these extended dietary patterns may affect breast milk composition, including vitamin C content [28]. This highlights the importance of culturally sensitive antenatal guidance on balanced confinement diets.

Study limitations

This study has several limitations that should be considered, with suggestions for future research where appropriate. First, vitamin C was analysed only at two time points, limiting the ability to observe changes across the full postpartum period. Previous research has shown that milk vitamin C can fluctuate across lactation stages [3], highlighting the need for more frequent sampling to better characterise its temporal pattern. Second, although breast milk samples were stored at -80°C in line with standard recommendations, the extended storage duration may have affected vitamin C stability, given its sensitivity to light, heat, and prolonged storage. Future research should aim to analyse samples within one to two years to preserve nutrient integrity. Additionally, changes in dietary intake were assessed using an ordinal food list questionnaire, which does not capture quantitative intake of vitamin C-rich foods. More detailed dietary assessment methods, such as 24-hour recalls or weighed food records, would enable a clearer understanding of the relationship between actual vitamin C intake and breast milk concentrations. Fourth, the analysis was based on a relatively small subset of mothers with adequate samples at both time points, and the findings should therefore be interpreted as exploratory. Finally, the study population was predominantly Malay and urban, which may limit the generalizability of the findings to other ethnic groups or rural populations in Malaysia. Despite these limitations, this study provides a meaningful foundation for examining the impact of culturally influenced postpartum diets on breast milk nutrient composition and underscores the need for culturally tailored nutrition interventions during the postpartum period.

## Conclusions

This study found that breast milk vitamin C concentrations among Malaysian mothers were lower than values reported in other populations. Vitamin C levels increased significantly from week 2 to weeks 8-12 postpartum, although concentrations at the two time points were strongly correlated, indicating consistent relative levels between individuals. Many mothers adhered to confinement dietary practices shaped by cultural beliefs, some of which involved restricting foods that may reduce overall dietary variety. The lower vitamin C concentrations observed at week 2 may be partly related to early postpartum confinement practices, which can limit intake of vitamin C-rich foods. Importantly, higher fruit intake during early confinement was associated with higher breast milk vitamin C concentrations, although a proportion of mothers reported reducing their intake of fruits and vegetables during confinement. These findings suggest that maternal nutrition education during the perinatal period should include practical guidance on maintaining adequate intake of vitamin C-rich foods, particularly fruits, during confinement. Such education could be integrated into antenatal counselling or postnatal care, using culturally appropriate messaging that respects traditional beliefs while promoting dietary balance. Future studies should involve larger, diverse populations and incorporate more frequent sampling across lactation.

## Appendices

Appendix A 

**Table 4:** Questionnaire on postpartum dietary changes Question to participants: During the postpartum period, have you made any changes to your diet for the following foods?

Food categories	Eat about the same	Did not eat before/now	Eat more	Eat less	Totally avoid	Specific food
Milk or dairy products						
Eggs & poultry						
Fish or seafood						
Nuts & peanuts						
Fruits						
Vegetables						
Vitamin / mineral supplements						
Herbal supplements						
Alcoholic drinks						
Others:						
